# Digital hypertension management: clinical and cost outcomes of a pilot implementation of the OMRON hypertension management platform

**DOI:** 10.3389/fdgth.2023.1128553

**Published:** 2023-09-20

**Authors:** Ericka C. Holmstrand, Hironori Sato, Jim Li, Abhishek Mukherjee, Nicole E. Fitzpatrick, Kenneth R. Rayl, Francis R. Colangelo

**Affiliations:** ^1^ VITAL Innovation Program, Highmark Health, Pittsburgh, PA, United States; ^2^ Technology Development HQ, Omron Healthcare Co., Ltd., Kyoto, Japan; ^3^ Premier Medical Associates, Allegheny Health Network, Monroeville, PA, United States

**Keywords:** hypertension, remote patient monitoring, blood pressure, cardiovascular risk, home monitoring device

## Abstract

**Importance:**

Home monitoring of blood pressure (BP) in hypertensive patients can improve outcomes, but challenges to both patient compliance and the effective transmission of home BP readings to physicians can limit the extent to which physicians can use this information to improve care. The OMRON Hypertension Management Platform (OMRON HMP) pairs a home BP cuff with a digital product that tracks data, provides reminders to improve patient compliance, and provides a streamlined source of information to physicians.

**Objective:**

The primary objective of the quality improvement (QI) project was to test the hypothesis that use of the OMRON HMP could reduce the number and cost of hypertension related claims, relative to a retrospectively matched cohort of insured members. A secondary objective was to demonstrate improvement in control of BP among patients.

**Design:**

Eligible members were recruited to the QI project between December 1, 2018 and December 30, 2020 and data collected for six months following recruitment. All members received the OMRON HMP intervention.

**Setting:**

Enrollment and data collection were coordinated on-site at selected PCP partner providers in Western Pennsylvania. Eligible members were identified from insurance claims data as those receiving care for primary hypertension from participating primary care physicians and/or cardiologists.

**Participants:**

Eligible members were between the ages of 35 and 85, with a diagnosis of primary hypertension. The retrospective cohort was selected from electronic medical records of Highmark-insured patients with hypertension who received care at Allegheny Health Network (AHN), a subsidiary of Highmark Health. Members were matched on baseline BP and lipid measures, age, smoking status, diabetes status, race and sex.

**Intervention:**

Daily home BP readings were recorded by the OMRON HMP app. Patient data was reviewed by clinical staff on a weekly basis and treatment plans could be adjusted in response to this data.

**Results:**

OMRON HMP users showed a significant increase in the number and cost of hypertension-related claims, contrary to the hypothesis, but did display improvements in control of BP.

**Conclusions and Relevance:**

The use of a digital platform to facilitate at-home BP monitoring appeared to improve BP control but led to increased hypertension-related costs in the short-term.

## Introduction

Primary hypertension affects over 100 million adults in the US and is a leading contributing cause to cardiovascular complications (1, 2). Among patients with diagnosed hypertension, those with masked uncontrolled hypertension (MUCH) may have in-office blood pressure readings within normal ranges, but experience higher pressures outside of the clinic setting (3). MUCH patients may comprise over 50% of the hypertensive population in the United States; these patients have suboptimal treatment outcomes that go undetected (4–6). MUCH patients are at increased risk of organ damage and cardiovascular disease compared to patients with controlled hypertension (5, 7).

Self-monitoring of blood pressure at home has been shown to be more effective than in-office measurement alone for the detection of patients with MUCH and for management of hypertension to reduce blood pressure (BP) with medication (8–11). Although plentiful evidence exists that at-home BP measurement improves outcomes in controlled trials, compliance with self-monitoring and medication adherence presents an ongoing challenge to achieving hypertension control (9, 10). Adherence to monitoring and treatment can be improved through the use of telemonitoring, in turn improving both the clinical outcomes and cost-effectiveness of self-monitoring (11–16).

Highmark Health is an integrated finance and delivery system (IFDS) headquartered in Western Pennsylvania that owns and operates both a large insurer (Highmark) and a regional hospital network [Allegheny Health Network (AHN)]. Highmark Health performs quality improvement studies of promising health interventions through its VITAL organization, making use of the combined access to member claims and medical records data afforded by its status as an IFDS. The OMRON hypertension management platform (HMP) pairs an FDA-cleared medical-grade home blood pressure monitor with a smartphone app that helps patients measure and record their blood pressure, provides medication reminders, and provides easy access to patient data by their primary care professional. A QI project was conducted in a population of insured members utilizing partner medical practices, to determine whether use of the OMRON HMP could (1) increase compliance with daily self-monitoring of blood pressure, (2) improve control of blood pressure, and (3) decrease health-care services utilization and cost. The primary hypothesis tested was that OMRON HMP test group members would have fewer hypertension-related medical claims incurred within the six months following enrollment than a comparison group drawn from Highmark Health's membership.

## Methods

The quality improvement test recruited participants from patients treated at partner clinics providing both PCP and specialist care to Western Pennsylvanians. Patient eligibility was determined from Highmark historical claims data and confirmed by the clinical staff through provider electronic medical records (EMR). The comparison group was identified from Allegheny Health Network (AHN) electronic medical records and Highmark's member and claims databases. In both groups, members were considered for inclusion if they were between 35 and 84 years of age and had an insurance claim indicating treatment for primary hypertension. Patients were excluded if they had any of the following conditions: pregnancy, active treatment for cancer, dementia, psychiatric conditions (see Supplementary Table S1 for full list of excluded conditions).

Test group members were also confirmed at the time of enrollment to be taking anti-hypertensive medication, willing to participate, and technologically capable of completing the test (i.e., using a mobile device capable of supporting OMRON HMP). This project was reviewed by the local Institutional Review Board (IRB) and was deemed as a QI initiative and not human subjects research. Therefore, the project did not fall under the IRB's purview and informed consent was not required. However, each member who decided to participate in this QI project was given a patient Participation Form informing them about the project in greater detail. The QI project was conducted between December 2018 and December 2020.

Recruited patients were included in the claims cost and utilization analysis only if they maintained continuous enrollment in a Highmark Commercial or Medicare Advantage insurance product for 6 months following their enrollment date.

A control group of Highmark members were drawn from the population of all Highmark members treated at AHN during the same timeframe as the Omron HMP group. Baseline demographic and biophysical data were collected from the AHN EMR database and the Highmark claims database for potential control group members: sex, race, diabetes status, smoking status, HDL, LDL, diastolic BP, systolic BP, and chronological age. The resulting pool of control group members was used to identify propensity- score matched controls for each test participant. The propensity-score matching procedure (PROC PSMATCH, SAS 9.4) was used to balance the distribution of baseline covariates: gender, race, diabetes status and smoking status were specified as exact matches, while all other covariates were matched within a range of −0.25 to +0.25 standardized mean difference. The optimal matching method within PROC PSMATCH resulted in a non-convergent model: the optimal variable ratio method was used instead.

A two-staged approach was necessary to match the maximum number of members. The first stage of propensity score matching was done on the full pool of 15,750 control group members to identify potential matches for 193 test group members. Based on optimal propensity score algorithm the potential control group list dropped to 1,062 potential matches from 15,750. Inclusion and exclusion criteria were then applied to this pool of potential matches using Highmark claims history, reducing the number of potential control group members to 624. A second round of PSMATCH was then applied, resulting in 579 potential control group members and 157 test group members for whom at least one match was identified by the procedure. Test group members with more than one match identified by the PROC PSMATCH procedure were subsequently matched to only one control group member by project personnel (KR).

After this first round of matching, 43 test group members remained who either had no matches identified from the first stage or were enrolled after the initial round of matching. These test group members were subjected to PSMATCH using a restricted pool of potential matches that excluded the 579 potential control group members identified as matches in the first round.

37 test group members were matched through the second round of propensity score matching. Supplementary Figure S1 shows the standardized mean differences for the test and control groups prior to and following the matching procedure.

For the medical cost and utilization analysis, an index date was set for each test and control group member. For test group members, the index date was equal to the date immediately following the enrollment date in the OMRON Hypertension Management Platform test. For control group members, the index date was set to the same date as their matched test group member. Continuous enrollment in a Highmark insurance product (excluding Medigap and other supplementary plans) for six months following the index date was required for inclusion in the claims' analysis. If either member of the matched pair did not maintain continuous coverage for the entire six-month post-enrollment period, then both individuals were excluded; re-matching was not performed.

All medical and pharmacy claims incurred by the test and control members during the six- month measurement period and adjudicated by Highmark within 90 days of the end of the measurement period were included in the dataset.

Medical and pharmacy costs represent the allowable charges determined by Highmark for each claim. Total medical costs over the six-month measurement period were aggregated and compared; in addition, sub-categories of costs related to hypertension diagnosis, cardiovascular care, and emergency room visits are reported. These claims were identified using either diagnosis codes (ICD-10-CM codes) associated with each claim or the associated setting of care. Pharmacy costs are reported as total costs of all medications over the six-month measurement period, as well as anti-hypertensive medication costs. See Supplementary Table S2 for included diagnosis, procedure, and prescription medication lists used to define the subcategories. In addition to cost metrics, the total number of claims submitted related to hypertension, emergency room, or cardiovascular care were compared between test and control groups.

Medical and pharmacy cost measures were compared between the propensity-score matched groups using the Wilcoxon two-sample test, using the two-sided probability and with significance set at the *α* = 0.05 level. The number of hypertension-related claims, and the number of cardiovascular-condition related claims were compared between groups using a zero-inflated Poisson regression model with a log link and a zeromodel utilizing a logit link. All data aggregation and statistical testing were conducted in SAS (SAS 9.4, Cary, NC).

## Results

204 Highmark members were recruited for the quality improvement test.

**Figure 1:**
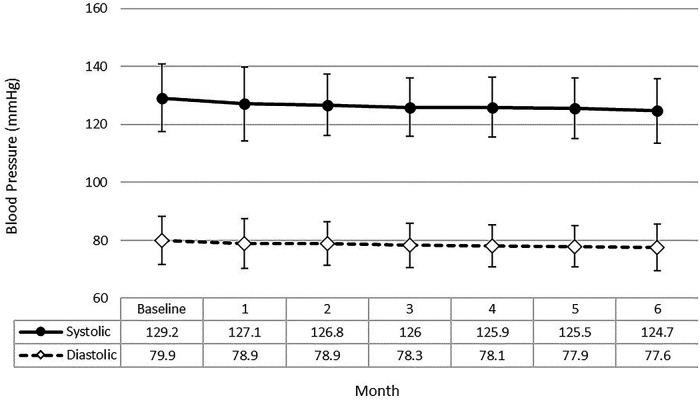
Average systolic and diastolic BP for OMRON test group participants over the course of the VITAL test. The mean values for the QI project population (*n* = 159) appeared to decline slightly but steadily over the six-month time period examined. Error bars show the standard deviation of the mean.

**Figure 2:**
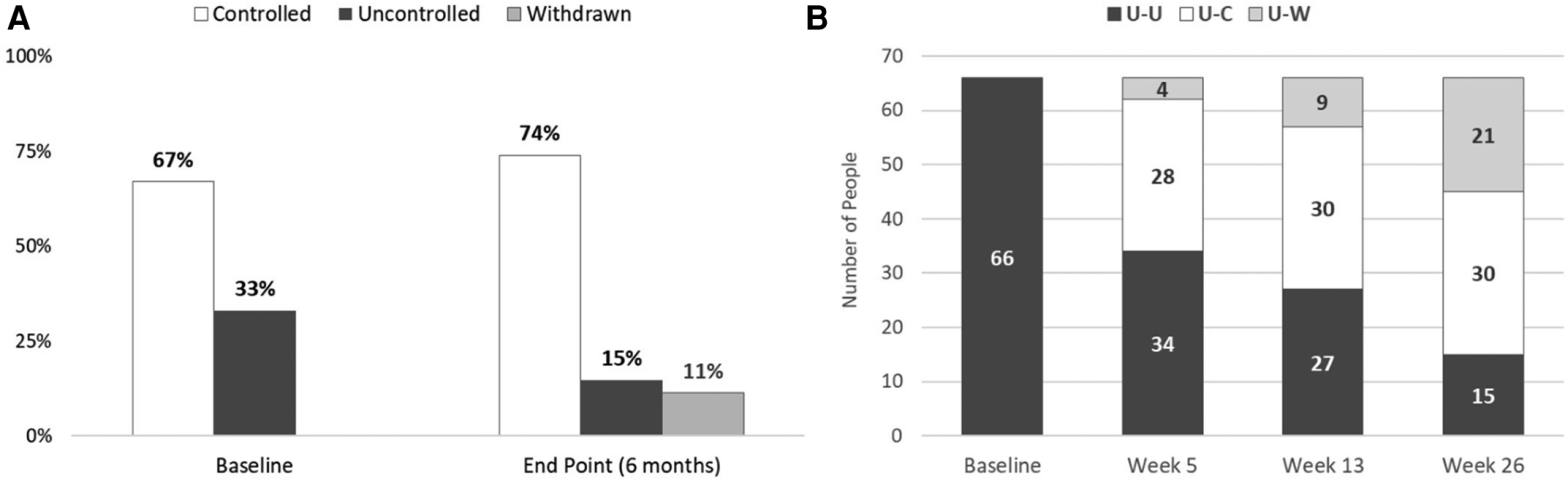
Baseline and endpoint characterization of patients’ status as uncontrolled or controlled hypertension. (**A**) The percentage of subjects with controlled and uncontrolled blood pressure is shown for the baseline at home BP readings, and at the conclusion of the QI project. For the end- point measurement, the last self-monitoring measurement recorded with the OMRON blood pressure monitor was used. The percentage of participants with controlled blood pressure (systolic <130 mm Hg and diastolic bp ≤ 80 mm Hg) increased from 67% to 74% of the sample population. (**B**) Test subjects who were identified as having uncontrolled hypertension at baseline were tracked to determine if they achieved blood pressure control over the course of the test. Of the 66 members initially identified as having uncontrolled hypertension at baseline, 30 (45%) were controlled at week 26. Most subjects who were able to achieve controlled status did so by week 5 of the QI project. U-U: uncontrolled at baseline, uncontrolled at endpoint; U-C: uncontrolled at baseline, controlled at endpoint; U-W: uncontrolled at baseline, withdrawn before endpoint.

Figure 1 shows the mean systolic and diastolic blood pressures for the 159 members who had BP measurements recorded by the OMRON HMP app at baseline and for each post- enrollment month. Over the course of the test, both systolic and diastolic pressures declined steadily. At baseline, one third of the participating members were characterized as having uncontrolled blood pressure; at the conclusion of the six-month QI project only 15% of the test group was characterized as uncontrolled (Figure 2A). Furthermore, the transition from uncontrolled to controlled status occurred within the first five weeks for most participants (Figure 2B).

Test members self-reported the frequency of their monitoring at home. At baseline, only 8% of test subjects monitored their blood pressure on a daily basis, but this increased to 50% at the six-month endpoint. Finally, test members reported high satisfaction levels with the OMRON HMP, with more than 85% of subjects somewhat or extremely satisfied on both three- and six- month post-enrollment surveys.

Provider experience and feedback regarding the OMRON HMP was collected through surveys and in-depth interviews. There were two themes that emerged from this feedback: (1) Clinicians believed the OMRON HMP would fit into their current clinical workflow and (2) the role of reviewing the data within the platform daily would be best suited for a nurse coordinator or similar role. The nurse would be responsible for reviewing any changes to a patient's status and informing the physician or advanced level practitioner of these changes for any medical decision making. In addition, the nurse coordinator could use the OMRON HMP to provide a summary of a patient's hypertension status to the physician or the advanced practitioner prior to a patient's standard of care office visit.

Data from 187 propensity score matched pairs were included in the medical claims analysis; 177 matched pairs were also included in the pharmacy claims analysis. Treatment group identity was a significant predictor of the number of medical claims incurred over the QI project period, with OMRON test group members incurring more hypertension-related claims (Parameter estimate 0.63, Wald *Χ*^2^ = 24.32, *p* < 0.0001), and more cardiovascular-condition related claims (Parameter estimate = 0.3252, Wald *Χ*^2^ = 5.78, *p* = 0.0163), than control group members.

Aggregate allowed medical costs incurred by the OMRON test group and control group are shown in Table 1. Both the total incurred for each group over the entire six-month measurement period and per member per month (PMPM) costs are shown for total medical cost and for the medical cost subcategories indicated.

**Table 1:** Medical and pharmacy claims’ costs.

	Control group		OMRON test group		% Difference in PMPM	*p*-value
	Total	PMPM	Total	PMPM		
All medical claims	$918,429	$819	$586,235	$522	−36%	0.1115
*Hypertension-related*	$13,947	$12	$18,130	$16	30%	<0.0001
*Cardiovascular-related*	$167,551	$149	$89,088	$79	−47%	0.4702
*Emergency room*	$248,835	$222	$38,226	$34	−85%	0.2747
*CV ER*	$72,176	$64	$28,434	$25	−61%	0.6361
All pharmacy claims	$276,893	$261	$175,168	$165	−37%	0.0108
*Hypertension drugs*	$11,735	$11	$8,416	$8	*−28%*	*0*.*0558*

Claims incurred by OMRON test group members and their matched controls in the six-month post-enrollment period are summarized in the table above. Total columns show the sum over the entire group and time period; PMPM columns are per member per month. *P*-values were obtained from two-sided two-sample Wilcoxon rank-sum tests and were considered significant at the *α* = 0.05 level.

The OMRON test group incurred significantly higher per member costs for claims with an indicated diagnosis of hypertension (Wilcoxon two-sample test, *Z* = 4.0269, *p* < 0.0001). Despite the lower allowed medical costs incurred overall for the OMRON test group, the cost difference between the matched pairs was not significant (*p* = 0.1115). Differences in all other medical claims cost categories were also apparently, but not significantly, lower for test group members. Removal of individuals or pairs with total costs greater than 3 standard deviations from their respective group mean (i.e., outlier removal) did not qualitatively alter these results.

Prescription drug costs were evaluated using the 177 matched pairs with continuous prescription drug coverage. OMRON test group members incurred lower total per member costs than the matched control group (Wilcoxon two sample test, *Z* = −2.5637, *p* = 0.0108). For anti- hypertensive drugs, the two groups were not significantly different (Wilcoxon two sample test, *Z* = −1.9189, *p* = 0.0558)

## Discussion

The results of the current test indicate that the OMRON Hypertension Management Platform is an engaging technology solution that helps patients with hypertension better manage their blood pressure. The integration of a blood pressure monitoring device with a smartphone app that can automatically record resulting data, generate medication and monitoring reminders, and provide real-time data to physicians led to improvements in blood pressure management for patients who had uncontrolled hypertension at the onset of the QI project.

Patients with MUCH are at increased risk of cardiovascular events (5) and may receive suboptimal care, as the incompleteness of their response to treatment goes undetected at clinic visits. The existence and prevalence of this subgroup demonstrates the importance of patient cooperation in regular home monitoring of blood pressure to achieve better condition management. Our QI project importantly identified a sizeable population of patients with MUCH and demonstrated improvement in these patients' blood pressure over the course of the QI project.

There is broad consensus that self-monitoring of BP at home can improve outcomes in the clinical management of hypertension (1, 17, 18), although a call for randomized controlled trials also persist (17). In addition, the ability for clinicians to review patients' home BP readings and use that information to titrate anti-hypertensive medication between clinic visits improves the timeliness of care. Other studies have suggested that active participation of a patient's medical team is central to achieving the best results (8, 11, 19, 20); however, the additional demands on physician engagement or other staff can erode cost-effectiveness (12) and even present a barrier to successful integration of home BP monitoring data into a patient's treatment plan (14). The addition of telemonitoring with an artificial intelligence (AI) based coaching function did not produce statistically significant improvement over a control group provided with a home BP monitor and tracking app without any coaching function (16). Our QI project made use of intermediary personnel to review member BP readings and alert physicians to the need for greater scrutiny and condition management, a compromise that appeared to support effective clinical implementation of the intervention, as significant improvement for those patients with uncontrolled hypertension was achieved.

The results of the claims cost analyses, by contrast, did not indicate that direct cost savings for total medical claims in the six months following enrollment could be attributed to participation in the test. The lack of statistical significance of the apparent cost savings for the OMRON test group was likely affected by the high variability of medical costs observed, and by the relatively small sample size examined. In addition, we examined claims costs only within a six-month timeframe. Increased home monitoring of BP is expected to result in medication adjustments, and possibly an increase in office visits to modify patients' treatment plans in the short term. As such, patients' higher utilization and cost profiles might be dominated by these early interventional visits. However, long term improvement in hypertension would eventually be expected to decrease overall medical services utilization, especially of high-cost services such as hospitalization for hypertension-related complications, and it is possible this could become evident at much later time points. In our analysis, costs for hypertension related claims in the test group did appear to rise over the first four months of the QI project, before decreasing in the last two months. Finally, the direct costs of the intervention itself were not included in our cost analysis, further complicating the demonstration of cost-effectiveness within the short timeframe of the QI project.

With a longer time period for analysis, and/or more widespread adoption of the OMRON HMP, significant medical cost savings might be demonstrated. Prior studies with similarly short timeframes have found that while telemonitoring of hypertension patients is cost-effective, this is established based on an acceptable demonstrated cost per quality adjusted life year (QALY), rather than a strict cost-savings model (11, 20). This implies that intervention participants in these studies did incur greater costs than control group members subject to usual care (i.e., clinic BP monitoring only). One study did demonstrate apparent cost-savings if the cost of the intervention were not included in the analysis, but, as with our QI project, this finding was not statistically significant (21). Interestingly, the per member per month (pmpm) direct medical cost savings was similar to our results ($281 vs. $297 reported herein). To our knowledge, few long- term studies (e.g., more than 2 years of monitoring) have been conducted to evaluate whether medical cost-savings are achieved as a result of avoiding hypertension-related disease complications and the resulting utilization of costly medical services to treat sequelae of uncontrolled hypertension, although clinical effectiveness has been demonstrated up to 2 years (19). A review of the economics of self-monitoring of blood pressure found mixed results; although most studies supported cost-effectiveness of SMBP at a threshold of <$50,000 per QALY, others failed to find an economic benefit, particularly of interventions that utilized the team-based care demonstrated to be crucial to clinical success (20).

As connected health devices are increasingly adopted into clinical management practices, continued investigation of the short- and long-term financial impacts of these technologies will be possible. Furthermore, improvements to the physician or clinician interface that decrease the variability in use of SMBP measurements and the administrative burden on clinic staff should yield better cost-effectiveness of SMBP-based interventions (22).

## Limitations

The main strength of this study was the combination of clinical data, patient experience, and economic data (from administrative claims data) in the same patient cohort allowing a comprehensive assessment of the impact of the Omron HMP. The main limitation was that randomization was not feasible, and while we utilized propensity score matching to control for key variables, there is still a potential for bias. Secondly, the HMP was offered to patients only at select clinical sites while the control group was derived from the broader Highmark membership. Thus, the control patient population is likely to be more heterogenous in terms of geography, and possibly other demographic characteristics. Third, this was designed as a real-world study and inclusion/exclusion criteria were purposefully broad. While this allowed a more realistic view of the impact of the HMP, it also could increase variability and uncertainly in the findings. Finally, a longer follow-up period would be beneficial to investigate the long-term impact of the platform.

## Data Availability

Due to the sensitivity of medical claims data, requests for sharing of de-identified datasets used for this analysis will be considered on a case-by-case basis by Highmark Health's Data Governance body. All requests should be directed to Ericka C. Holmstrand at ericka.holmstrand@highmarkhealth.org
