# Supplementary material for: Digital hypertension management: clinical and cost outcomes of a pilot implementation of the OMRON hypertension management platform
Source: Front Digit Health. 2023 Sep 20;5:1128553. doi: 10.3389/fdgth.2023.1128553 (PMC10548242; doi:10.3389/fdgth.2023.1128553)
Supplement: Supplementary file 1 [file Table1.docx]

**Supplementary Table S1 Excluded Conditions**

The table shows the medical conditions and associated insurance claims diagnostic and/or procedural codes that disqualified potential subjects or control group members. ICD-10-CM codes shown with only two digits indicate that all subcodes were included in the exclusion criteria (i.e. I21 includes I21.0, I21.01, etc).

| **Condition** | **ICD10-CM Codes** | **CPT/HCPCS Codes** |
| --- | --- | --- |
| Secondary Hypertension | I15.0, I15.1, I15.2, I15.8, I15.9 |  |
| Hypertensive Urgency | I16.0, I16.1, I16.9 |  |
| Pregnancy | Z31, Z32, Z33, Z34, Z35, Z36, all ‘O’ codes |  |
| Arrythmia | I47, I48, I49 |  |
| Dialysis treatment | Z49 |  |
| Recent Myocardial Infarction (within past year) | I21, I22, I23 |  |
| Pacemaker | Z95.0, Z45.0, Z95.810 | 0387T, 0388T, 0389T, 0390T, 0391T |
| Cardiovascular Conditions | I20, I24, I25, I46, I50, I60, I61, I62, I63, I64, I65, I63, I67, I68, I69, I71 | 92920, 92921, 92924, 92925, 92928, 92929, 92933, 92934, 92937, 92938, 92941, 92943, 92944 |
| Psychosis & Other Mental Health Disorders | F06, F07, F09, F10, F11, F12, F13, F14, F15, F16, F17, F18, F19, F20, F21, F23, F24, F25, F28, F29, F30, F31, F32, F33, F34, F39, F40, F41, F42, F43, F44, F45, F48, F50, F51, F52, F53, F54, F55, F59, F60, F63, F64, F65, F66, F68, F69, F70, F71, F72, F73, F78, F79, F80, F81, F82, F84, F88, F89, F90, F91, F93, F94, F95, F98, F99 |  |
| Dementia | F00, F01, F02, F03, F04, F05 |  |
| Cancer | D37, D38, D39, D40, D41, D42, D43, D44, D45, D46, D47, D48, Z08, Z85 |  |
