# Supplementary material for: Digital hypertension management: clinical and cost outcomes of a pilot implementation of the OMRON hypertension management platform
Source: Front Digit Health. 2023 Sep 20;5:1128553. doi: 10.3389/fdgth.2023.1128553 (PMC10548242; doi:10.3389/fdgth.2023.1128553)
Supplement: Supplementary file 2 [file Table2.docx]

**Supplementary Table S2**

**A Medical Claims Sub-categories**

The table shows the ICD-10-CM and/or other insurance claims codes used to classify medical costs incurred during the study period. Codes with “xx” shown indicate that all digits or characters in the corresponding positions were included in the category (i.e. I21.xx indicates that I21.0, I21.01, etc are all included).

| **Sub-Category** | **ICD-10-CM Codes** | **Other Codes** |
| --- | --- | --- |
| Hypertension related | I10.  I12.xx I13.xx  I16.xx |  |
| Cardiovascular (CV) related | I20.xx, I21.xx, I22.xx, I23.xx, I24.xx, I25.xx, I46.xx, I47.xx, I48.xx, I49.xx, I50.xx, I60.xx, I61.xx, I62.xx, I63.xx, I64.xx, I65.xx, I66.xx, I67.xx, I68.xx, I69.xx, I71.xx, R94.30, R94.31, R94.39, R00.0, R00.1,  R00.2, R00.8, R00.9, R01.1, R01.2, R93.1 | **CPT:** 929xx, 930xx, 932xx, 933xx, 934xx,  935xx, 936xx, 937xx  **ICD-10-PCS:** 02xxxxx, 03xxxxx, 04xxxxx, 05xxxxx, 06xxxxx, 4A02xxx, 4A12xxx, 5A02110, 5A02115, 5A02116, 5A0211D,  5A02210, 5A02115, 5A02216, 5A0221D,  5A12012, 5A1213Z, 5A1221Z, 5A1223Z,  5A2204Z, 4B02XTZ, 8E023DZ, B2xxxxx,  C2xxxxx  **DRG:** 215, 216, 217, 218, 219, 220, 221, 222,  223, 224, 225, 226, 227, 228, 229, 231, 232, 233,  234, 235, 236, 239, 240, 241, 242, 243, 244, 245,  246, 247, 248, 249, 250, 251, 252, 253, 254, 258,  259, 260, 261, 262, 263, 264, 265, 266, 267, 268,  269, 270, 271, 272, 273, 274, 280, 281, 282, 283,  284, 285, 286, 287, 288, 289, 290, 291, 292, 293,  294, 295, 296, 297, 298, 299, 300, 301, 302, 303,  304, 305, 308, 309, 310, 311, 313, 314, 315, 316,  319, 320  **Revenue codes** (Internal) for Coronary Care, Heart/Arterial X-ray, Cardiac Service, EKG or  Heart Transplant Service |
| Emergency Room (ER) |  | **BETOS** = M3  **Revenue Codes** (internal) for Emergency Room **Place of Service** (internal) for Emergency Room **Milliman Line Categories:**  FOP Emergency Care or Professional  Emergency/Observation care) |
| CV ER | Combined ER and Cardiovascular related flags | |

**B Hypertension-related Prescription Drugs**

| **Drug Name** |
| --- |
| AMLODIPINE BESYLATE CARTIA XT  DIGOXIN FELODIPINE ER NIFEDIPINE ER VERAPAMIL ER  AMLODIPINE BESYLATE-BE ATENOLOL  BISOPROLOL FUMARATE CANDESARTAN CILEXETIL CARVEDILOL  ENALAPRIL MALEATE HYDRALAZINE HCL IRBESARTAN LABETALOL HCL LISINOPRIL  LISINOPRIL-HYDROCHLORO LOSARTAN POTASSIUM LOSARTAN-HYDROCHLOROTH METOPROLOL SUCCINATE METOPROLOL TARTRATE OLMESARTAN MEDOXOMIL OLMESARTAN-HYDROCHLORO PROPRANOLOL HCL ER QUINAPRIL HCL  VALSARTAN  VALSARTAN-HYDROCHLOROT |
