# Supplementary material for: Digital hypertension management: clinical and cost outcomes of a pilot implementation of the OMRON hypertension management platform
Source: Front Digit Health. 2023 Sep 20;5:1128553. doi: 10.3389/fdgth.2023.1128553 (PMC10548242; doi:10.3389/fdgth.2023.1128553)
Supplement: Supplementary file 3 [file Image1.pdf]

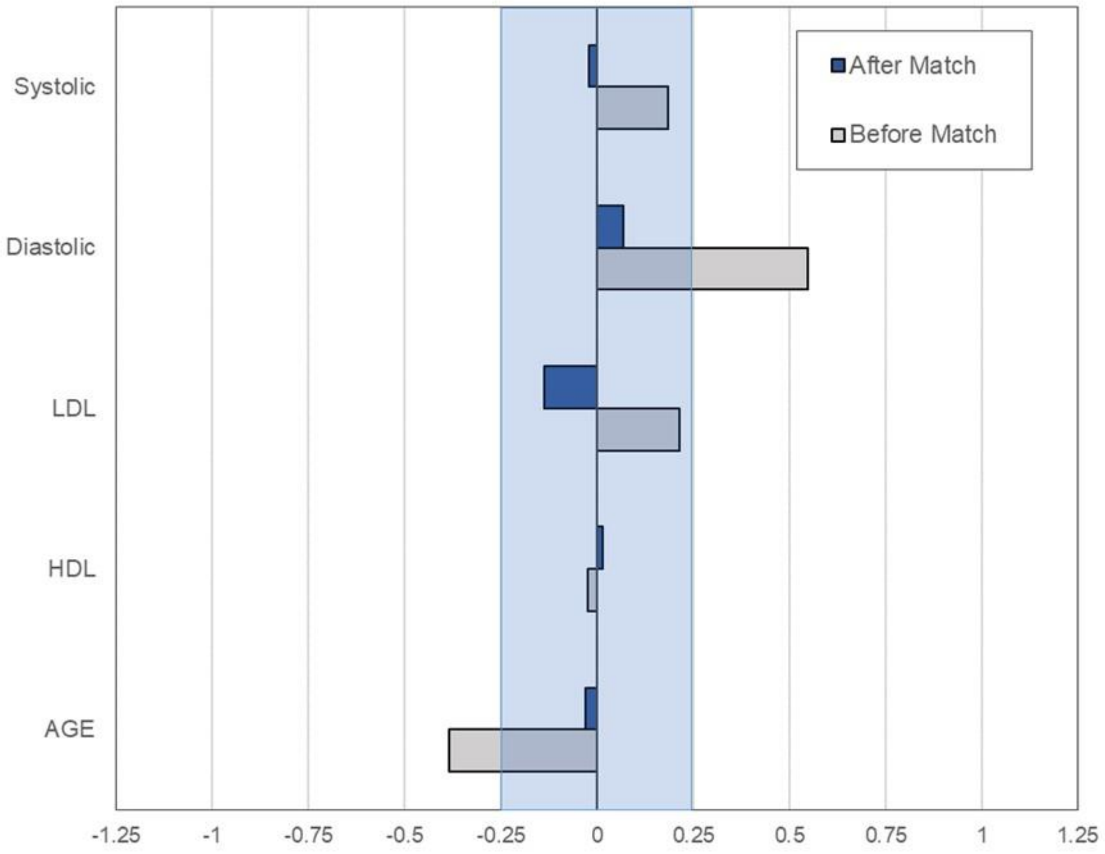

Supplemental Figure S1

The standard differences on selected variables prior to and after propensity score matching are shown in the figure above. The binary variables for which exact matching was used are not shown. Shaded area denotes the caliper setting used for acceptable matching. All variables used fell within the acceptable range of difference in the final matched pair populations used for the cost analysis.
